# Anti-Photoaging and Anti-Inflammatory Effects of Ginsenoside Rk3 During Exposure to UV Irradiation

**DOI:** 10.3389/fphar.2021.716248

**Published:** 2021-10-04

**Authors:** Shichao Wan, Yannan Liu, Jingjing Shi, Daidi Fan, Binglin Li

**Affiliations:** ^1^ Shaanxi Key Laboratory of Degradable Biomedical Materials, School of Chemical Engineering, Northwest University, Xi’an, China; ^2^ Shaanxi R and D Center of Biomaterials and Fermentation Engineering, School of Chemical Engineering, Northwest University, Xi’an, China; ^3^ Biotech and Biomed Research Institute, Northwest University, Xi’an, China; ^4^ College of Food Science and Technology, Northwest University, Xi’an, China

**Keywords:** anti-photoaging, inhibiting inflammation, ginseng, skin, ginsenoside Rk3

## Abstract

Ginseng is a widely cultivated perennial plant in China and Korea. Ginsenoside Rk3 is one of the major active components of ginseng and is a promising candidate to regulate skin pigments and exert anti-photoaging effects on skin physiology. Ginsenoside Rk3 was mixed with a cream (G-Rk3 cream) and smeared on the skin of mice. Then, the mice were exposed to ultraviolet (UV) A (340 nm and 40 W) and UVB (313 nm and 40 W) radiation. Special attention was given to the anti-photoaging and anti-inflammatory effects of ginsenoside Rk3 on the mouse skin. Macroscopic evaluation indicated that the mouse dorsal skin looked smooth and plump even under UV irradiation for 12 weeks. Pathological analysis indicated that there was no obvious photoaging or inflammation in the mouse skin that was treated with the G-Rk3 cream. More healthy, intact, and neat collagen fibers were observed in mice treated with the G-Rk3 cream than in untreated mice. Further analysis proved that ginsenoside Rk3 could inhibit the decrease in water and hydroxyproline levels in skin tissues and the loss of superoxide dismutase and glutathione peroxidase activities in the blood. Moreover, ginsenoside Rk3 slowed or halted increases in malondialdehyde, matrix metalloproteinase (MMP)-1, and MMP-3 levels in the blood and levels of interleukin 1, interleukin 6, and tumor necrosis factor α in skin tissues. In conclusion, ginsenoside Rk3 plays a significant role in inhibiting photoaging and inflammation to protect skin health.

## Introduction

Human skin will be exposed to more solar ultraviolet (UV) radiation due to ozone-layer depletion (Gromkowska Kępka et al., 2021; Lyons et al., 2021). Although many scientists have tried their best to slow, halt, or reverse the destruction of the ozone layer, such as reducing the emission of chlorofluorocarbons, this recovery process requires several years or even decades (Krutmann and Berneburg, 2021). Therefore, we should focus on the use of the existing resources for treating or preventing the damage induced by UV radiation (Sambandan and Ratner, 2011; Huang and Chien, 2020). Photoaging is one of the main causes of damage from long-term UV radiation and is very hard to avoid in daily life (Poon et al., 2015). UV exposure causes skin wrinkling, photoaging, drying, roughness, lack of elasticity, and melanogenesis. In severe cases, it can even lead to skin cancer (Sander et al., 2021).

Animal experiments have shown that the skin of mice appeared wrinkled, thickened, and stained after exposure to UV radiation for a long time (Gromkowska Kępka et al., 2021). Sharp decreases were observed in skin elasticity, water content, and collagen content (measured by hydroxyproline (HYP)) (Petruk et al., 2018). Lipid oxidation is another important sign of body aging and consists of a highly complex set of free radical reactions (Petruk et al., 2018; Chen et al., 2020). Superoxide dismutase (SOD) and glutathione peroxidase (GSH-Px) are cytosolic enzymes that can reduce peroxide radicals and protect organisms from oxidative damage (Kammeyer and Luiten, 2015; Nanda and Madan, 2021). Therefore, skin health should be consistent with the activities of SOD and GSH-Px in the serum (Pandel et al., 2013; Nanda and Madan, 2021). In contrast, the level of malondialdehyde (MDA), which is a lipid oxidation product, is increased (Pandel et al., 2013). Matrix metalloproteinase (MMP) expression is another indicator of skin aging (Duan et al., 2019; Lee et al., 2021). High MMP expression accelerates the degradation of the extracellular matrix in the dermis and damages the connective tissue of the skin (Pittayapruek et al., 2016; Xiao et al., 2020). On the other hand, the skin, as an independent immune organ, is affected by UV radiation and generates inflammatory and immune reactions (Kandan et al., 2020). Interleukin 1 (IL-1), interleukin 6 (IL-6), and tumor necrosis factor α (TNF-α) are three important cytokines in the skin tissue that can initiate and regulate skin inflammation, leading to local inflammation in the skin (Lee et al., 2019; Ke and Wang, 2021). A previous study showed that UV radiation induces the generation of reactive oxygen species (ROS) and accelerates the oxidation of lipids, resulting in light damage to cells and DNA (Serafini et al., 2015; Brem et al., 2017). Moreover, the ROS also promote the synthesis and secretion of IL-1, IL-6, and TNF-α. Thus, the levels of IL-1, IL-6, and TNF-α can be used to reflect the degree of damage caused by UV radiation (Poon et al., 2015; Fuller, 2019).

Ginseng (*Panax ginseng* Meyer) is a beneficial herb that is a widely cultivated perennial plant in Korea and China and has been used for centuries to prevent and treat various diseases (Wong et al., 2015; Liu and Fan, 2019). Ginsenosides are major active components of ginseng (Wong et al., 2015). In skin physiology, ginsenosides are known to regulate pigments and exert antiaging effects (Nam et al., 2017; Liu et al., 2018). Ginsenoside Rg1 was found to increase melanogenesis and the tyrosinase activity in human melanocytes (Gao et al., 2010). In contrast, ginsenoside F1, which is the hydrolysis metabolite of Rg1, can inhibit visible pigmentation (Han et al., 2014). Ginsenosides consist of many components, such as ginsenosides 20(S)-Rg3, 20(R)-Rg3, Rk3, Rh4, Rk1, and Rg5 (Xu et al., 2016). Except for Rg1, the properties of other components have been less investigated. Ginsenoside Rk3 is a kind of nature active components of ginseng, which has been used as a functional food and medicine since 100 years in many countries (Xu et al., 2016).

In this study, special attention was given to the effects of the rare natural ginsenoside Rk3 on inhibiting photoaging and inflammation in the mouse skin induced by UV irradiation. Ginsenoside Rk3 was mixed with a cream that could be manufactured as a sunscreen. This cream was smeared on the mouse skin, which was then exposed to UVA and UVB radiation. Then, the health of the mouse skin was monitored and pathological analysis of mouse skin was conducted to examine the changes in collagen fibers. Several crucial parameters were measured to evaluate the anti-photoaging and anti-inflammatory properties of ginsenoside Rk3, including skin elasticity, water content, SOD and GSH-Px activities, and HYP, MDA, MMP, IL-1, IL-6, and TNF-α levels.

## Materials and Methods

### Materials

**FIGURE 1:**
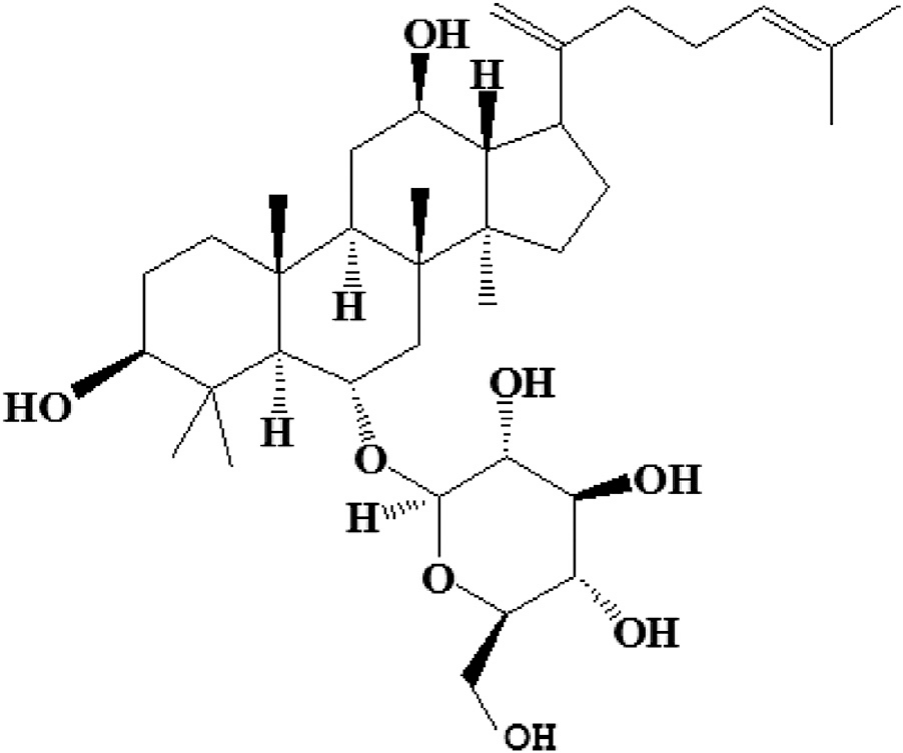
The chemical structure of ginsenoside Rk3.

Kunming mice were purchased from PLA Air Force Military Medical University (Xi’an, China). Ginsenoside Rk3 (≥98%) was obtained from Xi’an Giant Biogene Technology Co., Ltd. The relevant preparation process is described in our previous study (Qu et al., 2019). The chemical structure of ginsenoside Rk3 is shown in Figure 1. Ginsenoside Rk3 was mixed with a cream to be manufactured as a sunscreen. This product was launched by our group (Xi’an Giant Biogene Technology Co., Ltd.) and recorded as “Shan G Zhuang Wang Bei Zi 2019001533 (Chinese: 陕G妆网备字2019001533)” in China. Detailed information can be found in our published patent (CN 202010419961.1). In this study, the G-Rk3 cream was used to represent this launched sunscreen. Another sunscreen was prepared in the same way but without adding ginsenoside Rk3 (normal cream). A UVA lamp with 40 W with a main crest of 340 nm and a UVB lamp with 40 W with a main crest of 313 nm were purchased from Kunshan Laibang Instrument Company (China). The animal study was reviewed and approved by the Northwest University Animal Ethics Committee (NWN-AWC-20210357M).

### Photoaging in the Mouse Skin

The ultraviolet rays from the sun generally consist of the long-wave UVA and medium-wave UVB, but not short-wave UVC. Therefore, UVA and UVB were simultaneously used to simulate UV irradiation. Hair in a 3 × 4 cm^2^ area on the back of each mouse was removed. The irradiation distance was controlled at 10 cm. Exposure was performed once every 2 days during the first week and once each day during the other weeks. This irradiation process was repeated for 12 weeks. The health of the mice was monitored. If ulcers or blisters were observed, irradiation was paused for 2 days.

40 Kunming mice were divided into four groups. Before photoaging, all mice were smeared with stroke-physiological saline solution for 1 week. Group I was the control group, and the mice in this group were smeared with triple-distilled water and did not undergo any UV irradiation. Groups Ⅱ (photoaging model group), Ⅲ, and IV were smeared with triple-distilled water, normal cream, and G-Rk3 cream, respectively. Then, after 1 hour, all mice were irradiated. A universal testing machine (Instron Corporation, United States) was used to measure the elastic modulus of the mouse skin tissue. Pathological samples were sent to Air Force Military Medical University (Xi’an, China) for analysis. Enzyme-linked immunosorbent assay (ELISA) kits were used to measure the activities of SOD and GSH-Px and the levels of HYP, MDA, MMPs, IL-1, and IL-6.

### Evaluation of Recovery From Stretching

Before pinch testing, mice were anesthetized. The dorsal skin at the midline was picked up with fingers until the animal was lifted into the air and maintained for 3 s. Subsequently, the mice were placed on the ground. The recovery time for the skin to return to its initial state was recorded.

### Evaluation of the Water Content

The mice were sacrificed by cervical dislocation, and their dorsal skin (without subcutaneous fat) was immediately collected and stored at −20°C. The tissue was weighed before and after drying to calculate the water content in the skin.

### Evaluation of Lipid Peroxidation

Three important indicators were used to evaluate lipid peroxidation: SOD activity and the levels of GSH-Px and MDA. Orbital blood was collected from the mice. The collected blood was quickly centrifuged at 1°C and stored at −20°C. The SOD activity and the levels of GSH-Px and MDA were measured by relevant kits (Nanjing Jiancheng Bioengineering Institute, China).

### Preparation of Skin Homogenates

Mouse skin (0.5 g) was cryopreserved at −80°C and washed twice with a precooled physiological saline solution. Filter paper was used to clean the skin surface. The mouse skin was put into a precooled EP tube, and 4.5 ml of physiological saline solution was added. The homogenizer (HomoLab, FBF Co., Italy) was used at a low temperature. The obtained solution was checked to ensure that no intact cells were observed. The obtained homogenate was centrifuged at 1°C. The supernatant was collected for further analysis.

### MMP Levels

The supernatant of skin homogenates was used to analyze the levels of MMP-1 and MMP-3 using ELISA kits (Shanghai Enzyme-Linked Biotechnology Co., Ltd., China).

### Evaluation of Skin Collagen

HYP is one of the major components of skin collagen, accounting for approximately 13.5% of mammalian collagen. Generally, the level of HYP should be stable in the healthy state and this can be used to represent the level of collagen. HYP was measured using relevant HYP kits (Nanjing Jiancheng Bioengineering Institute, China).

### Evaluation of Skin Inflammation

IL-1, IL-6, and TNF-α are three important cytokines in the skin tissue (Lee et al., 2019). These cytokines are crucial triggers to initiate and regulate skin inflammation and were analyzed by ELISA (Shanghai Enzyme-Linked Biotechnology Co., Ltd., China).

For each parameter (involving skin elasticity, water content, the activities of SOD and GSH-Px, and the levels of HYP, MDA, MMPs, IL-1, IL-6, and TNF-α), the relative difference was calculated as follows:

$$Relative\;difference=\;\frac{group\;\text{X}-\;group\;\text{I }}{group\;\text{I}\;}\times \;100\text{\% }\text{.}$$ (1)

In Equation 1, group X represents the value of each parameter in groups Ⅱ, Ⅲ, or IV.

Improvement efficiency was identified as the crucial indicator to evaluate the normal cream and G-Rk3 cream.

The improvement efficiency of the normal cream was calculated as follows:

$$\text{Improvement}\;\text{efficiency}\;\text{of}\;\text{the normal cream}=\frac{group\text{II}-group\text{III}}{group\text{III}}\times 100\text{\%}\text{.}$$ (2)

In Equation 2, group II and group III represent the value of the relative difference of the parameter in each group.

The improvement efficiency of the G-Rk3 cream was calculated as follows:

$$\text{Improvement}\;\text{efficiency}\;\text{of}\;\text{the G}-\text{Rk}3\;\text{cream}=\frac{group\text{II}-group\text{IV}}{group\;\text{IV}\;}\times 100\text{\% }\text{.}$$ (3)

In Equation 3, group II and group IV represent the value of the relative difference of the parameter in each group.

The improvement efficiency of the G-Rk3 cream compared with the normal cream was calculated as follows:

$$\text{Improvement}\;\text{efficiency}\;\text{of}\frac{\text{G}-\text{Rk}3\text{ cream}}{\text{normal cream}}=\frac{group\text{III}-group\text{IV }}{group\text{IV}\;}\times 100\text{\%}\text{.}$$ (4)

In Equation 4, group III and group IV represent the value of the relative difference of the parameter in each group.

## Results and Discussion

### Macroscopic Evaluation of the Dorsal Skin

Aging is a slow process. The skin, which is the outermost barrier, plays a very important role in preventing the effects of ultraviolet rays, temperature, humidity, etc. (Lai-Cheong and Mcgrath, 2017). Among these factors, photoaging induced by UV irradiation is the most serious factor associated with skin aging (Brem et al., 2017). Therefore, the effects of ginsenoside Rk3 on inhibiting photoaging and inflammation were investigated.

**FIGURE 2:**
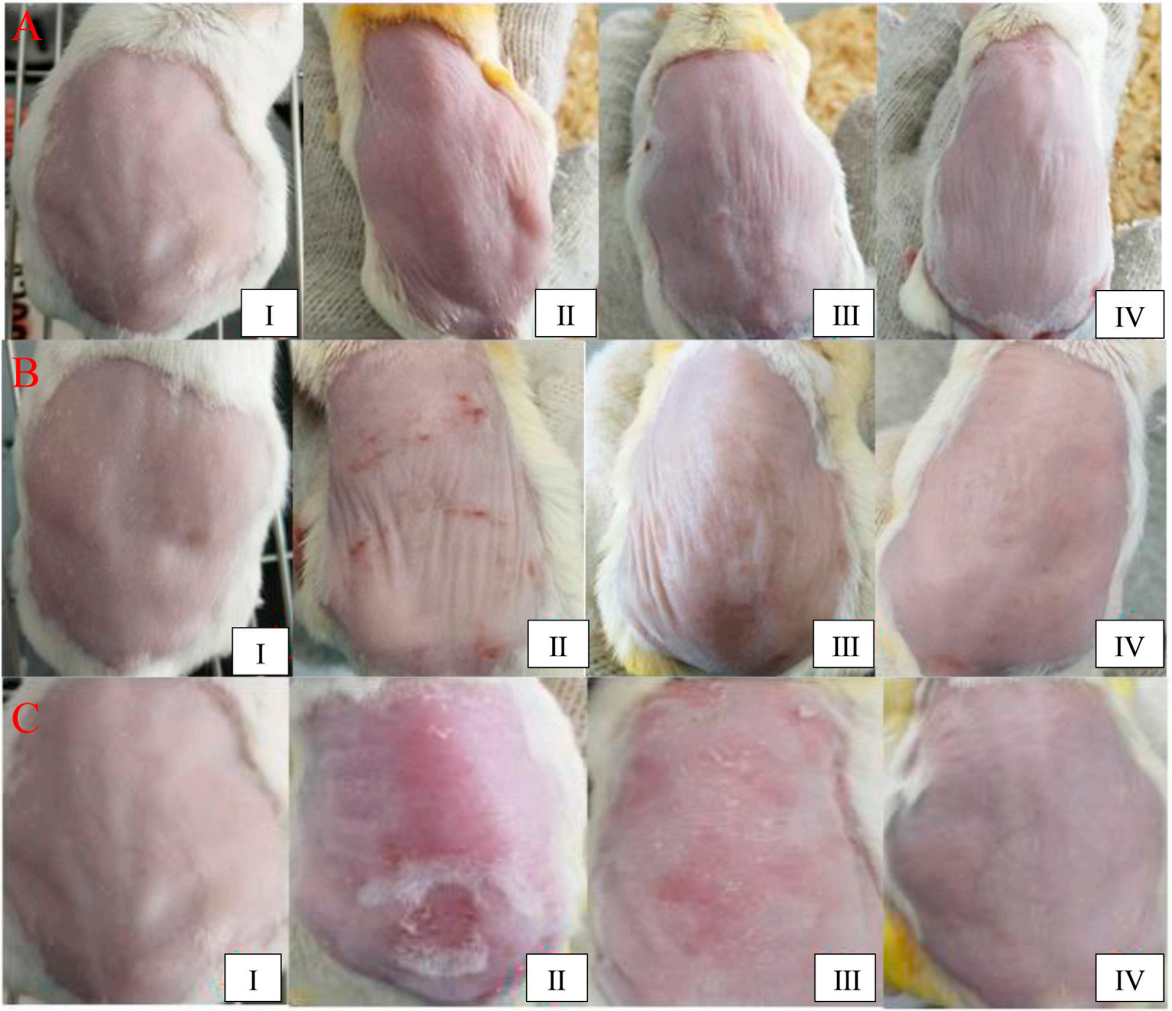
Macroscopic evaluation of the dorsal skin. UV irradiation for 2 weeks **(A)**; 6 weeks **(B)**; and 12 weeks **(C)**.

Figure 2A shows the control group, which represents healthy mouse skin. As shown in Figure 2B, the photoaging model was established by UV irradiation of the mouse skin tissue. After 2 weeks, wrinkles were observed in the skin in the photoaging model group and the skin showed slight redness and appeared sunken. Moreover, blood vessels were easier to identify than in other groups. After 6 weeks, obvious wrinkles and a sunken appearance were observed. The entire skin looked very dull. Clear sunburn marks were observed, which might have been induced by ruptured capillaries. After 12 weeks, the health of the mouse skin in group Ⅱ was very poor. Significant ulcers and blisters were observed in a large area of the skin. In group Ⅲ, the normal cream without ginsenoside Rk3 was used to prevent photoaging, as shown in Figure 2C. Compared with that of group Ⅱ, the health of the skin was improved slightly. After UV irradiation for 2 weeks, slight wrinkles and a sunken appearance were observed but no redness was observed. After 6 weeks, wrinkles and a sunken appearance were observed. The mouse skin gradually became dull and lost elasticity. Although no obvious sunburn marks were observed, blood vessels in the back became easy to identify in this stage. After 12 weeks, the health of the mouse skin in group Ⅲ was also very poor. A significant loss of the dermal tissue was observed by the naked eye. Severe ulcers and blisters were observed in a large area of the skin.

Although severe ulcers and blisters were observed in group Ⅱ, the health of the skin in group IV was improved dramatically by the use of the G-Rk3 cream. As shown in Figure 2D, the mouse skin looked smooth and plump even under UV irradiation for 12 weeks. Very few wrinkles were observed in all cases. In this group, no ulcers or blisters were observed; thus, UV irradiation was never paused. Theoretically, the irradiation time in group IV was the longest among the groups. Except for ginsenoside Rk3, the normal cream and G-Rk3 cream have the same ingredients. Therefore, ginsenoside Rk3 played a very effective role in inhibiting photoaging and inflammation.

### Evaluation of Recovery From Stretching

**FIGURE 3:**
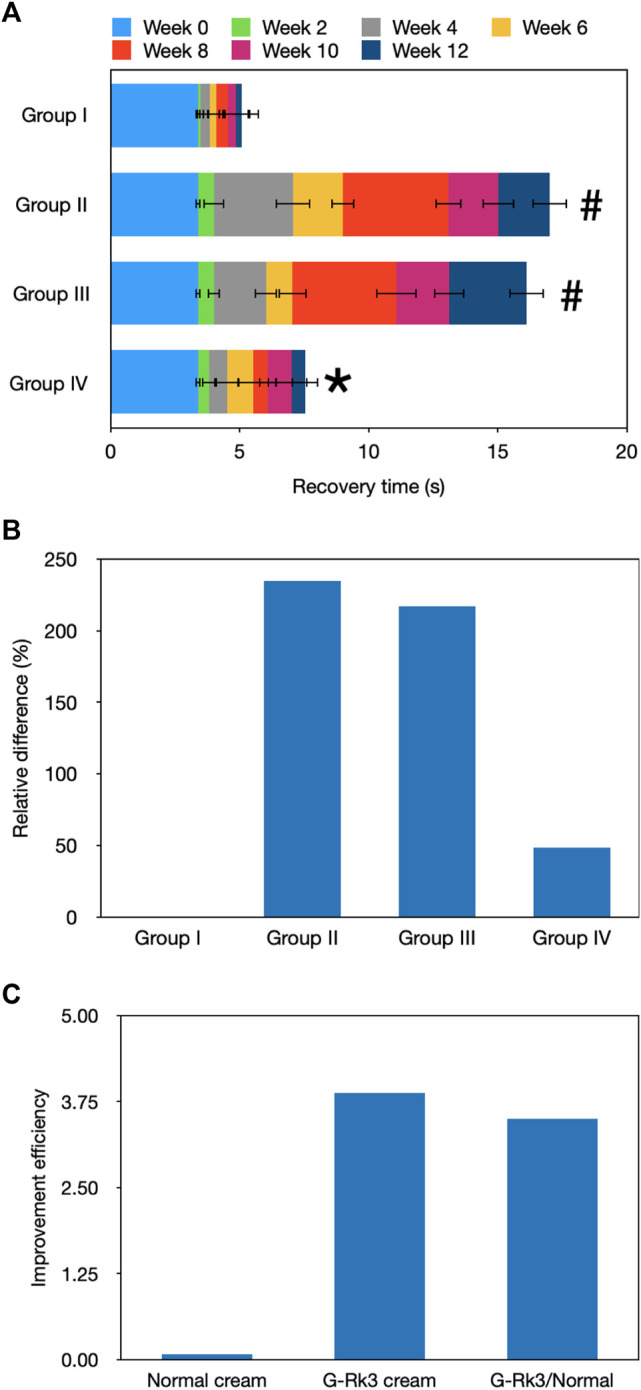
Evaluation of recovery from stretching. Recovery time **(A)**. The absolute value of the recovery time in the initial state (0 weeks) was used. In other weeks, only the increment per 2 weeks is shown. #Significantly different from group I, *p* < 0.05. *Not different from group I, *p* > 0.05. Relative differences in the four groups **(B)**. Improvement efficiencies of normal and G-Rk3 creams **(C)**.

Skin elasticity is a comprehensive indicator that reflects the health of the skin (Lai-Cheong and Mcgrath, 2017). Figure 3A shows the recovery time required by the mouse skin after lifting, which was an important indicator of photoaging. After UV irradiation for 2 weeks, a SPSS analysis indicated that there was no obvious difference among the four groups (*p* > 0.05). After 6 weeks, a significant difference was observed in group Ⅱ (*p* < 0.05), suggesting that a successful photoaging model group was established. Moreover, group Ⅲ also showed a significant difference compared with group I (*p* < 0.05) but no significant difference compared with group Ⅱ (*p* > 0.05). Although the normal cream slightly protected the mouse skin, it could not slow or halt the photoaging process, and the photoaging speeds in groups Ⅱ and Ⅲ were very similar (Figures 2, 3). Thus, the normal cream did not have anti-photoaging properties. After UV irradiation for 12 weeks, the photoaging degrees in groups Ⅱ and Ⅲ were very severe and they did not show any significant differences (*p* > 0.05), but their photoaging was significantly different from that in group I (*p* ≈ 0.018). As shown in Figure 3B, recovery time periods in these two groups were 235 and 217% higher than those in group I, respectively. Compared with that of triple-distilled water, the anti-photoaging effect of the normal cream was increased by 8%, as shown in Figure 3C. In contrast, the photoaging effects in groups I and IV did not show any significant differences (*p* > 0.05), indicating that skin elasticity was maintained by the administration of the G-Rk3 cream. The recovery time was only increased by 48%. The anti-photoaging effect of the G-Rk3 cream sharply increased by 79% compared with that of triple-distilled water, which was 3.5 times higher than that of the normal cream.

### Hematoxylin and Eosin Staining of the Mouse Skin

**FIGURE 4:**
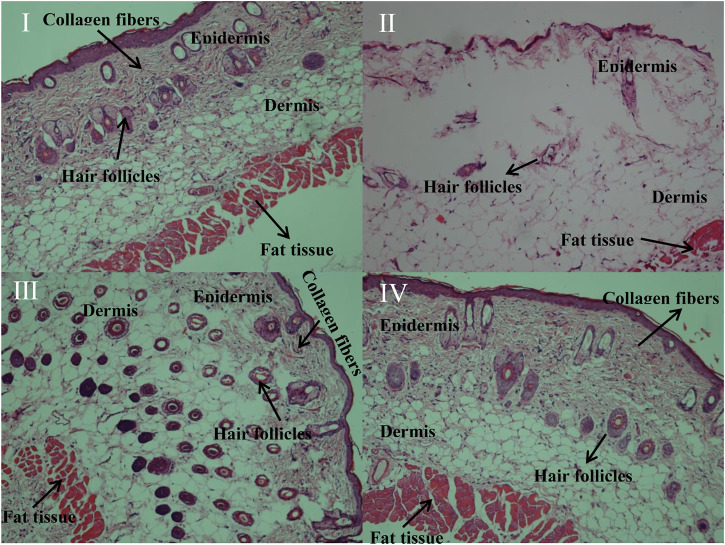
H&E staining of the mouse skin.

The histological hallmark of photoaging is dermal elastosis, which largely consists of thickened, tangled, and ultimately granular amorphous elastic structures (Rittié and Fisher, 2015). Long-term UV irradiation may damage dermal fibroblasts, leading to abnormal elastin or digestion of the extracellular matrix induced by inflammatory mediators (Kandan et al., 2020). The typical photoaged skin can be characterized by epidermal hyperplasia, dermal elastosis, matrix protein degradation, and perivenular lymphohistiocytic dermal infiltrates. After UV irradiation for 12 weeks, we examined the characteristic histological features of the epidermis and dermis. The hematoxylin and eosin (H&E) staining analysis of different groups is shown in Figure 4. Normal control group I and group IV showed similar features. The epidermis had multiple obvious layers of squamous cells, which were covered by a thin layer of keratin and certain stratum granulosum cells. A basement membrane was observed beneath the basal layer. Moreover, collagen fibers and elastic tissue fibers were concentrated on the superficial dermis. Clusters of sebaceous glands were attached to hair follicles, and sweat glands were mostly located on the superficial dermis. The deeper dermis showed abundant fat with regularly distributed hair follicles; vascular channels were also regularly distributed. The skin of mice that were administered with G-Rk3 cream (group IV) had a similar status to that in control group I. A slight difference was observed only in the basement membrane. Somewhat swollen and indistinctly fuzzy skin regions were observed in mice in group IV. However, no inflammation was detected. This pathological analysis indicated that ginsenoside Rk3, as an ingredient in the cream, could significantly reduce or nearly eliminate photoaging induced by UV irradiation.

The mouse skin in photoaging group Ⅱ was seriously burnt, and the epidermal thickness sharply decreased. The health of the mouse skin that was administered with the normal cream (group Ⅲ) was slightly improved but also showed features similar to those of group Ⅱ. Serious cell pyknosis was observed in the epidermal region. The epidermis showed acute thickening, which was associated with fuzziness of the basement membrane and merging into the dermal collagen. The stratum corneum was shed, exposing the epidermis, the junction of the epidermis and dermis flattened, and the dermal papilla and epidermal protrusions disappeared. Many elastic tissues and collagen fibers were destroyed, and the rest also showed extreme disorganization and distribution. Moreover, edema and inflammation were observed in the dermis between fat cells and hair follicles, and capillaries were congested or even broken. Thus, the normal cream did not protect the mouse skin from photoaging. These pathological results suggested that ginsenoside Rk3 was an efficient anti-photoaging ingredient to protect the skin from UV irradiation.

### Evaluation of the Water Content

**FIGURE 5:**
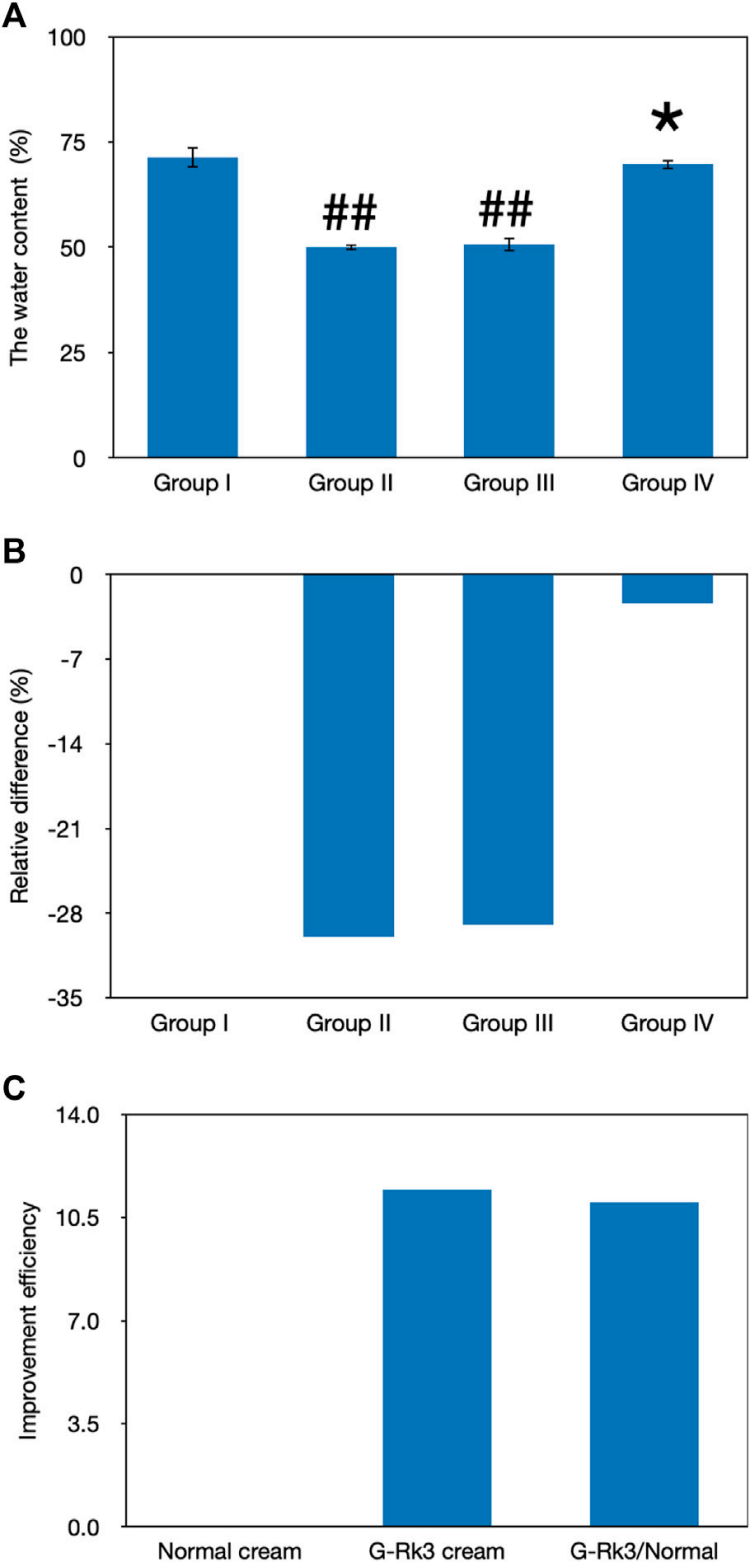
Evaluation of the water content. The absolute value of the water content **(A)**. ##Very significantly different from group I, *p* < 0.01. *Not different from group I, *p* > 0.05. Relative differences in the four groups **(B)**. Improvement efficiencies of normal and G-Rk3 creams **(C)**.

The water content in the skin tissue plays a very important role in maintaining skin elasticity (Pangestuti et al., 2021). The loss of the water content is one of the signs of aging, and UV irradiation can accelerate this process, leading to skin photoaging. As shown in Figure 5, the gravimetric method was used to analyze the water content in the skin tissues of mice in the four groups after UV irradiation for 12 weeks. Compared with that of group I, the water content in the skin in groups Ⅱ and Ⅲ decreased significantly (*p* < 0.01). The degree of decline in both these groups reached approximately 30%. However, the loss of water was only 2.4% in the mouse skin smeared with the G-Rk3 cream (group IV). This finding suggested that group IV showed no significant difference from group I (*p* > 0.05). Compared with that of triple-distilled water, the moisturizing efficiency of the normal cream was only increased by 3.2% but that of the G-Rk3 cream was sharply increased by 92%. Therefore, the moisturizing efficiency of the G-Rk3 cream was notably improved and was 28.5 times higher than that of the normal cream. The only different ingredient between the normal cream used in group Ⅲ and the G-Rk3 cream used in group IV was ginsenoside Rk3. Thus, ginsenoside Rk3 has excellent moisturizing properties for the skin.

### Evaluation of Lipid Peroxidation

**FIGURE 6:**
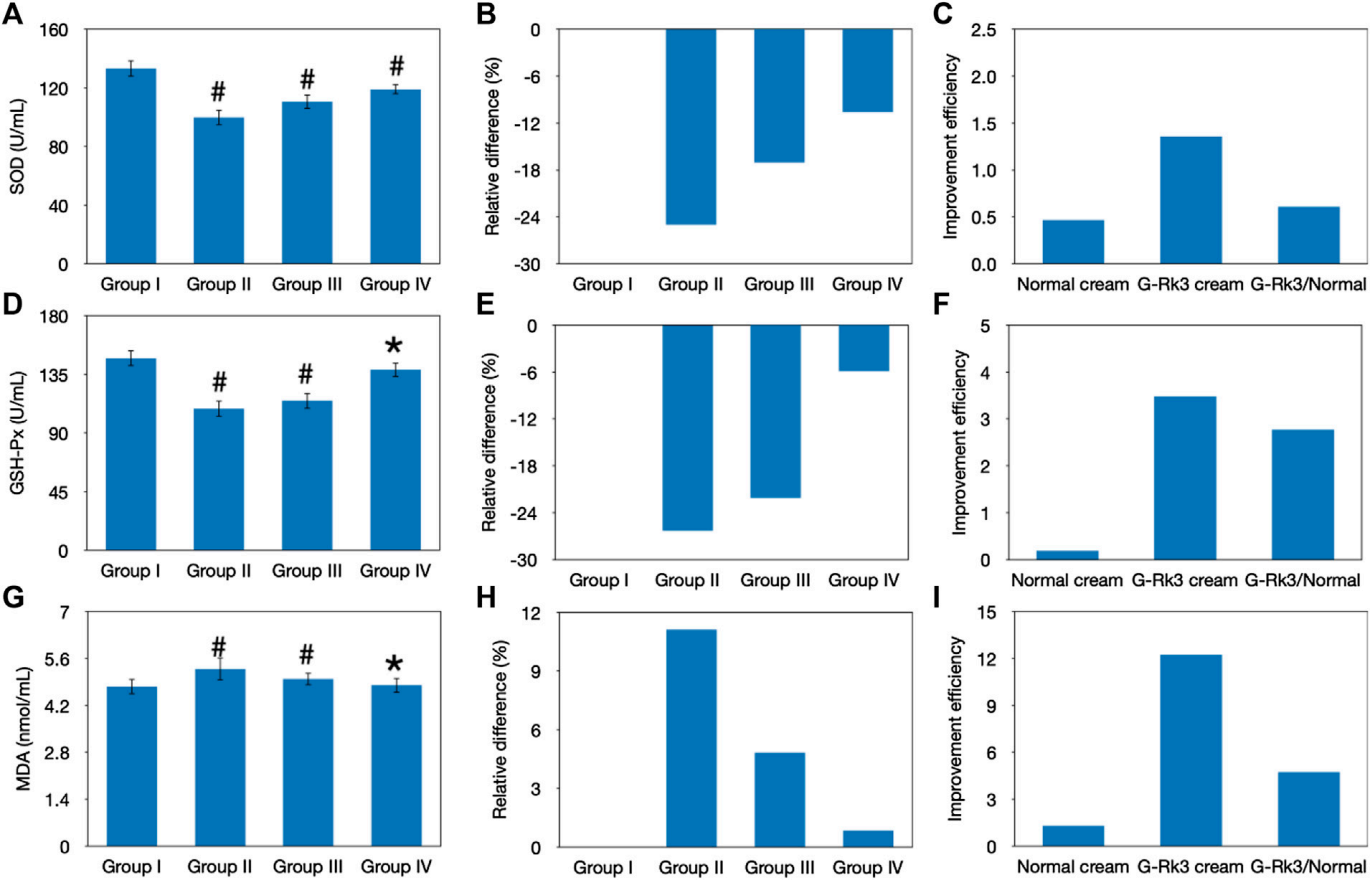
Evaluation of lipid peroxidation. Changes in the SOD activity **(A–C)**, GSH-Px activity **(D–F)**, and MDA concentration **(G–I)**. The absolute value **(A, D, G)**. #Significantly different from group I, *p* < 0.05. *Not different from group I, *p* > 0.05. Relative differences in the four groups **(B, E, H)**. Improvement efficiencies of normal and G-Rk3 creams **(C, F, I)**.

Lipid oxidation is another important sign of body aging and consists of a highly complex set of free radical reactions (Petruk et al., 2018; Chen et al., 2020). SOD, as an important antioxidant defense in nearly all living cells, can selectively catalyze the dismutation (or partitioning) of superoxide radicals into ordinary molecular oxygen and hydrogen peroxide (Kammeyer and Luiten, 2015; Nanda and Madan, 2021). Thus, the activity of SOD can be used to reflect free radical–scavenging ability and represents the antioxidant ability of the organism (Nanda and Madan, 2021). After 12 weeks of UV irradiation, changes in the activities of SOD in the four groups were examined and are shown in Figures 6A–C. Compared with that of control group I, a loss of SOD activity was observed in the other groups (*p* < 0.05), suggesting a decrease in the free radical–scavenging activity. The lowest activity was observed in mice in group Ⅱ, and the degree of decline reached 25%. Compared with that of triple-distilled water, protective effects of the normal cream and G-Rk3 cream against the loss of SOD activity were 32 and 58%, respectively. Therefore, the addition of ginsenoside Rk3 prevents the loss of SOD activity.

Superoxides are produced as a byproduct of oxygen metabolism and, if not regulated, can cause many types of cell damage (Serafini et al., 2015). GSH-Px, a peroxidase, can protect the organism from endogenously and exogenously induced lipid peroxidation (Pandel et al., 2013). Thus, GSH-Px activity represents the antioxidant ability of the organism. As shown in Figures 6D–F, a sharp decrease in GSH-Px activities was observed in groups Ⅱ and Ⅲ after UV irradiation for 12 weeks (*p* < 0.05). The degree of decline in these groups reached 26 and 22%, respectively; in contrast, that in group IV was only 5.9%, and no significant difference from group I was observed (*p* > 0.05). Thus, long-term UV irradiation had a significantly negative effect on GSH-Px activity, reducing the antioxidant ability of the skin tissue, but the normal cream barely prevented this aging effect. Interestingly, the effect of the G-Rk3 cream against the decrease in GSH-Px activity reached 78%, which was 4.9 times higher than that of the normal cream, suggesting the excellent antioxidant ability of ginsenoside Rk3.

MDA is one of the products of lipid oxidation (Dunaway et al., 2018). Thus, the residual concentration of MDA in the serum can reflect the peroxidation degree of lipids in the skin tissue (Dunaway et al., 2018). Figures 6G–I shows that UV irradiation increased the concentration of MDA by 11% in group Ⅱ (*p* < 0.05). Interestingly, the normal cream also showed acceptable inhibition of lipid oxidation in group Ⅲ (*p* > 0.05). When the normal cream was used instead of triple-distilled water, the concentration of MDA was increased by 4.8% and the improvement efficiency reached 57%. This effect might be explained by the fact that the normal cream could prevent the loss of SOD activity (Figure 6) and, thus, the increase in hydrogen peroxide, which is necessary for lipid oxidation, was slowed. Furthermore, the best performance was observed when the G-Rk3 cream was used because the process of lipid oxidation was almost halted (*p* > 0.05). Only a 0.8% increase in the MDA concentration was observed. The improvement efficiency of the G-Rk3 cream reached 92%, which was 1.6 times higher than that of the normal cream. The mechanism behind this effect might be that the G-Rk3 cream could efficiently prevent the loss of SOD and GSH-Px activities simultaneously. The increase in hydrogen peroxide was slowed, and its removal through reduction was also accelerated.

### Matrix Metalloproteinase Levels and Evaluation of Skin Collagen

**FIGURE 7:**
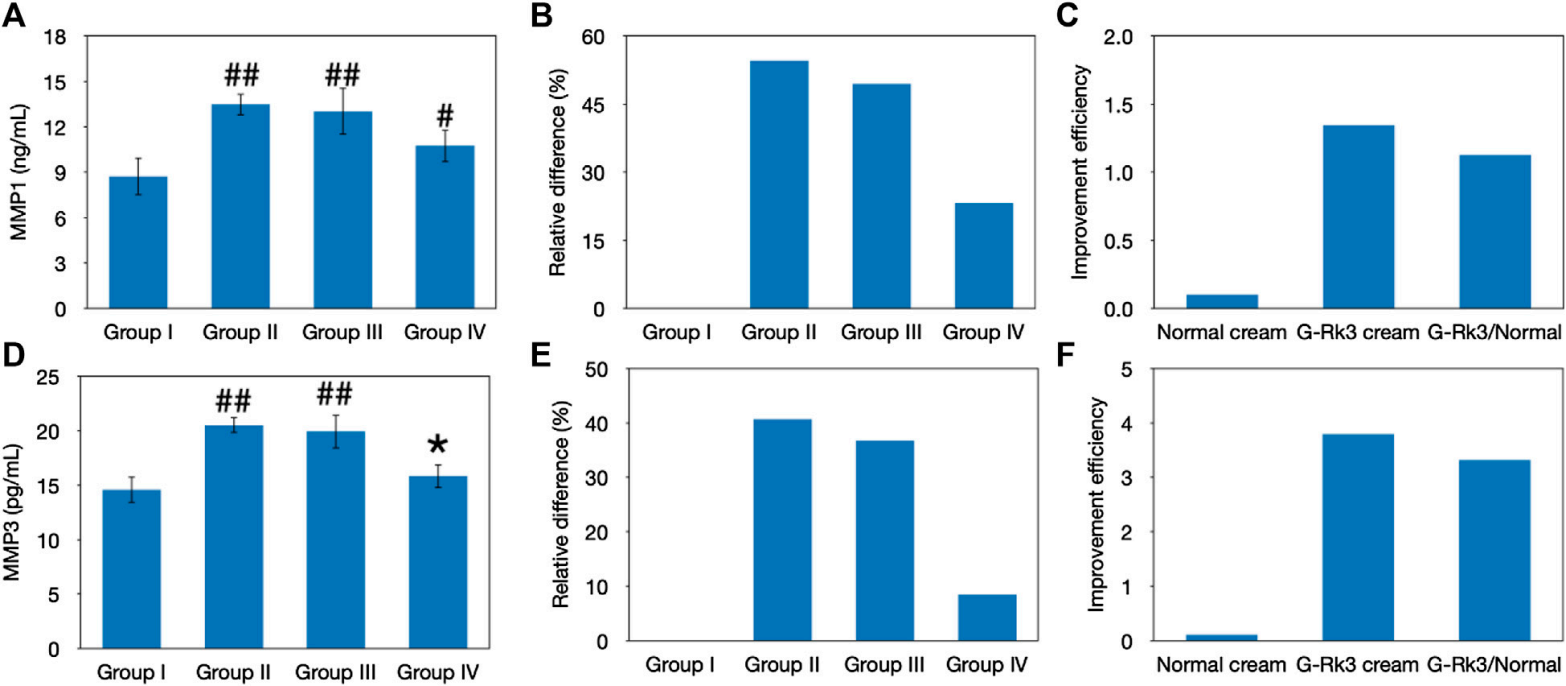
Evaluation of MMPs. Changes in the levels of MMP-1 **(A–C)** and MMP-3 **(D–F)**. The absolute value **(A, D)**. ##Very significantly different from group I, *p* < 0.01. #Significantly different from group I, *p* < 0.05. *Not different from group I, *p* > 0.05. Relative differences in the four groups **(B, E)**. Improvement efficiencies of normal and G-Rk3 creams **(C, F)**.

Previous studies indicated that MMPs can denature virtually all cartilage components (Duan et al., 2019; Lee et al., 2021). Thus, the abnormal expression of MMPs in the skin tissue will accelerate degradation of the extracellular matrix and damage connective tissues, making the skin thickness uniform, decreasing skin elasticity, and leading to wrinkling and other photoaging effects (Pittayapruek et al., 2016). ELISA kits were used to analyze MMP-1 and MMP-3, as shown in Figure 7. The results indicated that UV irradiation stimulated the expression of these MMPs. When the G-Rk3 cream was smeared on the back of mice in group IV, the expression of MMP-1 (*p* > 0.01) and MMP-3 (*p* > 0.05) was increased by only 23 and 8.5%, respectively. These results indicated that the effect of the normal cream on inhibiting MMP overexpression was only approximately 9% compared with that of triple-distilled water. However, the G-Rk3 cream inhibited MMP-1 and MMP-3 by 57 and 79%, respectively. The addition of ginsenoside Rk3 to the normal cream increased its ability to inhibit MMP-1 and MMP-3 by 6.2- and 8.1-fold, respectively.

**FIGURE 8:**
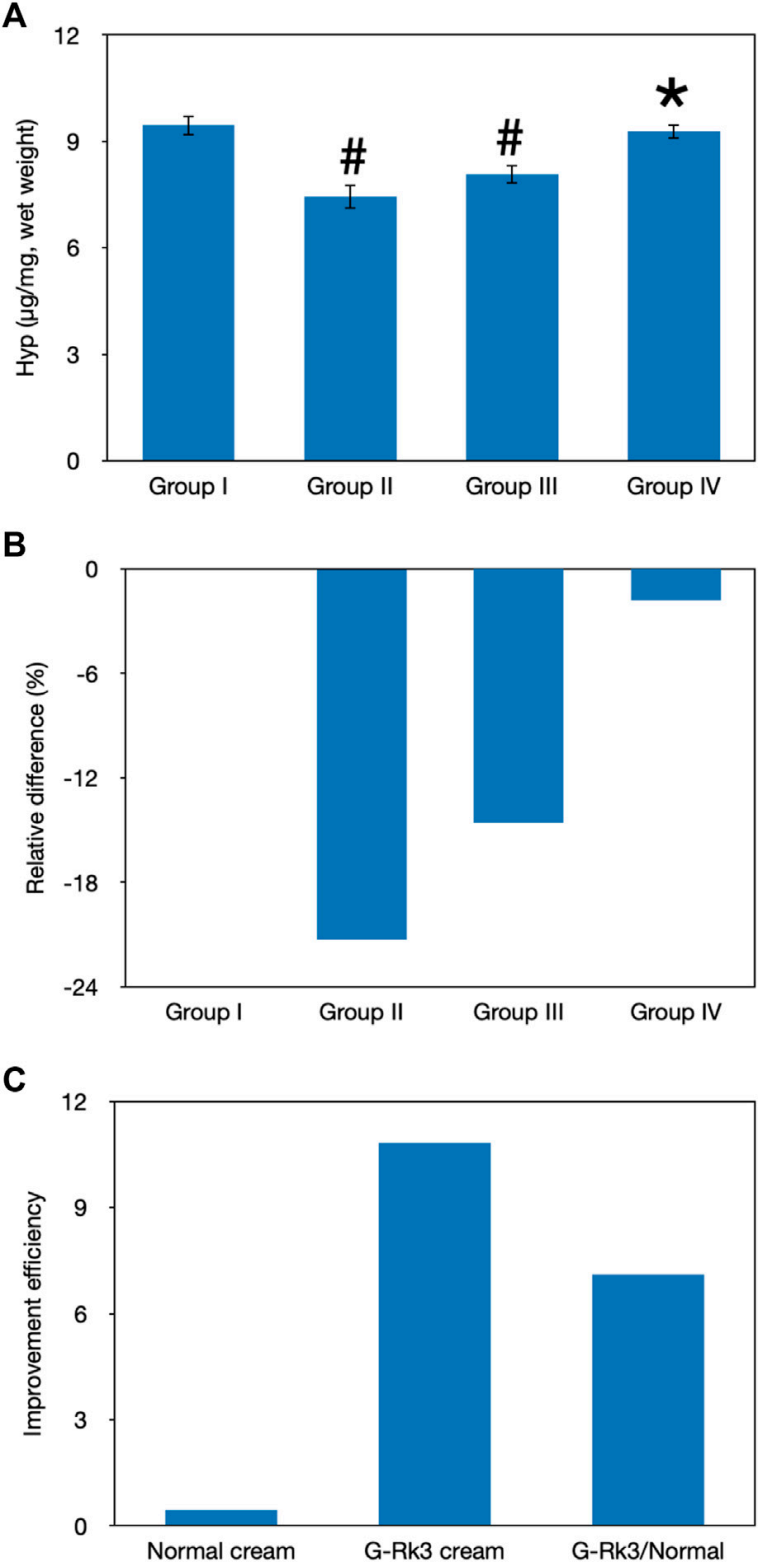
Evaluation of skin collagen. The absolute value of HYP levels **(A)**. #Significantly different from group I, *p* < 0.05. *Not different from group I, *p* > 0.05. Relative differences in the four groups **(B)**. Improvement efficiencies of normal and G-Rk3 creams **(C)**.

HYP is one of the major components of skin collagen and accounts for approximately 13.5% of mammalian collagen (Shin et al., 2019). The main function of HYP is to permit the sharp twisting of the collagen helix (Shin et al., 2019). Generally, the level of HYP should be stable in the healthy state and this can be used to represent the level of collagen (Shin et al., 2019). As shown in Figure 8, the HYP content in photoaging model group Ⅱ and group Ⅲ was different from that in control group I (*p* < 0.05). In contrast, the statistical analysis indicated that there was no difference between group IV and group I (*p* > 0.05). Compared with that of mice administered with triple-distilled water, the HYP content of mice administered with the normal cream (group Ⅲ) improved by 31%, while that of mice administered with the G-Rk3 cream (group IV) increased by 92%. Thus, the prevention of HYP loss was improved by 2.9 times when ginsenoside Rk3 was added to the normal cream. The above results demonstrated that G-Rk3 significantly decreased MMP levels and increased the HYP content, as the hallmarks of photoaging in skin collagen.

### Evaluation of Skin Inflammation

**FIGURE 9:**
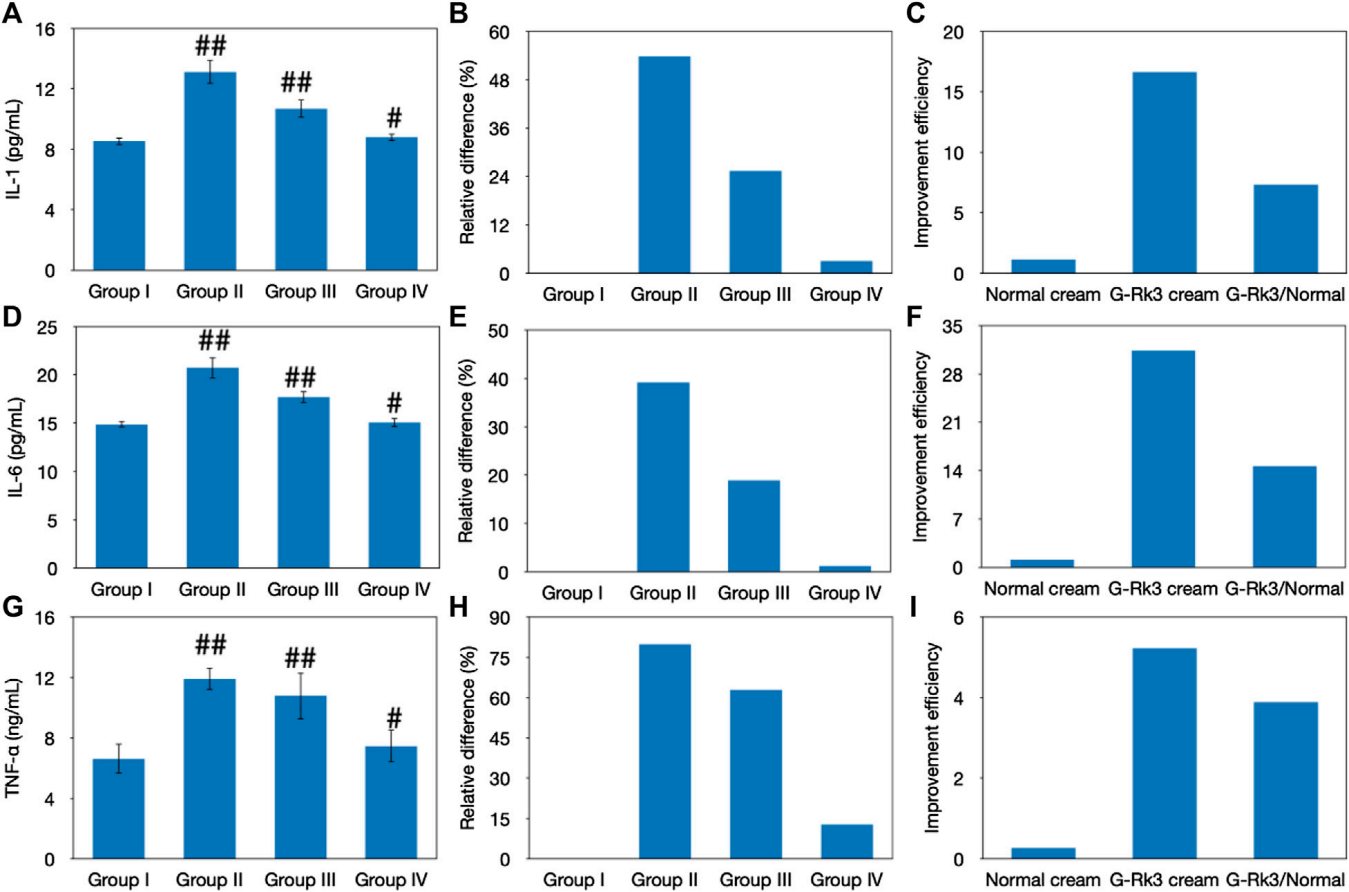
Evaluation of skin inflammation. Changes in the levels of IL-1 **(A–C)**, IL-6 **(D–F)**, and TNF-α **(G–I)**. The absolute value **(A, D, G)**. ##Very significantly different from group I, *p* < 0.01. #Significantly different from group I, *p* < 0.05. Relative differences in four groups **(B, E, H)**. Improvement efficiencies of normal and G-Rk3 creams **(C, F, I)**.

Previous studies have proven that UV irradiation promotes the secretion of IL-1, IL-6, and TNF-α, which are three important cytokines in the skin tissue (Fuller, 2019; Lee et al., 2019). These cytokines are the crucial triggers that initiate and regulate skin inflammation and form an important part of the inflammatory response of the body against infection. By existing on the cell surface and in intracellular compartments, these factors can increase the expression of adhesion factors and activate the transmigration of immunocompetent cells, such as phagocytes and lymphocytes, to sites of infection. The synergistic effect among IL-1, IL-6, and TNF-α generally causes fever, hyperalgesia, vasodilation, and hypotension. As shown in Figure 9, the experimental results indicated that the concentrations of IL-1, IL-6, and TNF-α in all groups were different from those in control group I (*p* < 0.05). The concentrations of IL-1, IL-6, and TNF-α in group Ⅱ increased by 54, 39, and 80%, respectively. An improvement was observed in the sham control group (group III), and the concentrations of IL-1, IL-6, and TNF-α increased by 25, 19, and 63%, respectively. However, the photoaging model (group Ⅱ) and sham control (group Ⅲ) were both significantly different from the control (group I) (*p* < 0.01). In contrast, only 3.1, 1.2, and 13% increases were observed in the concentrations of IL-1, IL-6, and TNF-α in group IV, respectively, which was not significantly different from those in group I (*p* > 0.01). These results explain why no obvious redness or allergy was observed in mice administered with the G-Rk3 cream in group IV (Figures 2, 4). Compared with that of triple-distilled water, the effectiveness of the normal cream to control the excessive secretion of IL-1 and IL-6 was improved by approximately 52%, but that of mice administered with the G-Rk3 cream (group IV) increased by approximately 96%. Moreover, the effect of the normal cream in controlling the excessive secretion of TNF-α was only improved by 21%, but that of mice administered with the G-Rk3 cream (group IV) increased by 84%. The secretion of IL-1 and IL-6 induced by UV irradiation was prevented by approximately 1.8 times when ginsenoside Rk3 was added to the normal cream. However, significant improvements were observed in preventing the secretion of TNF-α when the G-Rk3 cream was used. The relevant effect was increased by 3.9 times higher than that of the normal cream.

## Conclusion

**FIGURE 10:**
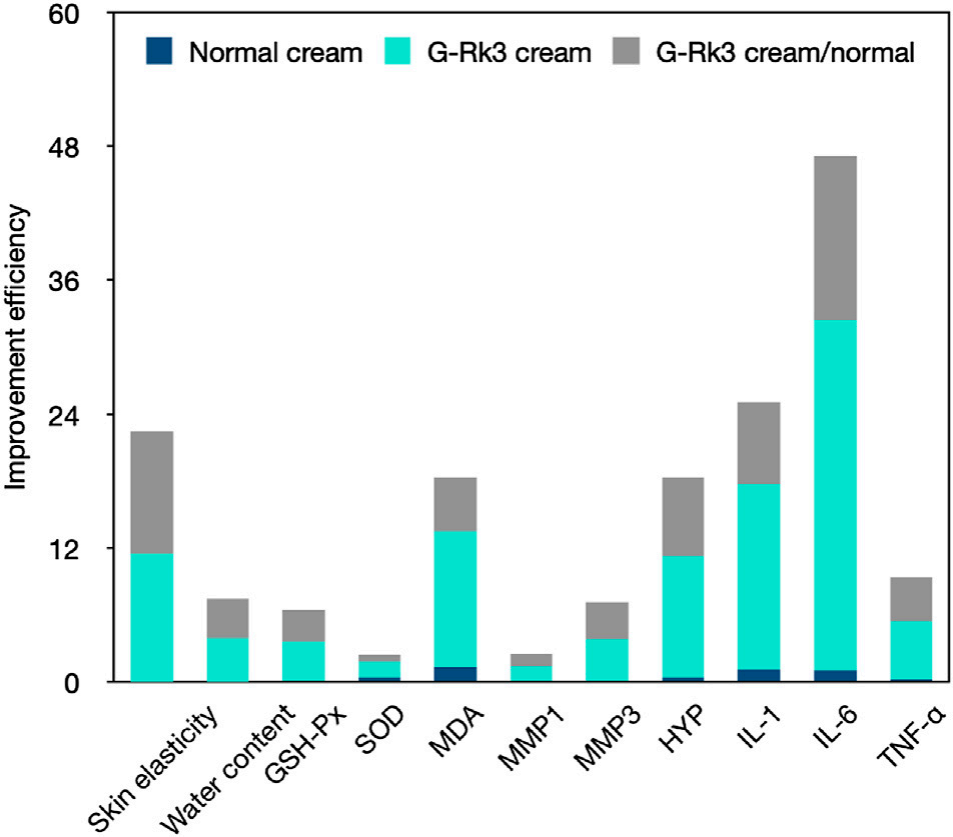
Comprehensive comparison of the anti-photoaging effects of the G-Rk3 cream and normal cream.

In conclusion, ginsenoside Rk3 could promote skin elasticity, inhibit the decrease in water and HYP levels in the skin tissue, and also increase the loss of SOD and GSH-Px activities in the blood. Moreover, ginsenoside Rk3 slowed or halted increases in MDA, MMP-1, and MMP-3 levels in the blood and IL-1, IL-6, and TNF-α levels in the skin tissue (Figure 10). The results of the present study illustrate that ginsenoside Rk3 has strong anti-photoaging and inflammation properties to protect skin health.

## Data Availability

The raw data supporting the conclusion of this article will be made available by the authors, without undue reservation.
